# Research on Spatial Localization Method of Magnetic Nanoparticle Samples Based on Second Harmonic Waves

**DOI:** 10.3390/mi15101218

**Published:** 2024-09-30

**Authors:** Zheyan Wang, Ping Huang, Fuyin Zheng, Hongli Yu, Yue Li, Zhichuan Qiu, Lingke Gai, Zhiyao Liu, Shi Bai

**Affiliations:** School of Information Science and Engineering, Shenyang University of Technology, Shenyang 110870, China; zheyan_edu@163.com (Z.W.); zhengfuyin666@163.com (F.Z.); 15774472716@163.com (H.Y.); ginobitu@163.com (Y.L.); qiuzc@smail.sut.edu.cn (Z.Q.); gailingke@smail.sut.edu.cn (L.G.); l13840566559@163.com (Z.L.)

**Keywords:** magnetic nanoparticle, Langevin function, DC bias field, second harmonic, tomographic positioning

## Abstract

Existing magnetic tracer detection systems primarily rely on fundamental wave signal acquisition using non-differential sensor configurations. These sensors are highly susceptible to external interference and lack tomographic localization capabilities, hindering their clinical application. To address these limitations, this paper presents a novel method for achieving the deep spatial localization of tracers. The method exploits second harmonic signal detection at non-zero field points. By considering the combined nonlinear characteristics of the coil’s axial spatial magnetic field distribution and the Langevin function, a correlation model linking the signal peak and bias field is established. This model enables the determination of the tracer’s precise spatial location. Building on this framework, a handheld device for localizing magnetic nanoparticle tracers was developed. The device harnesses the second harmonic response generated by coupling an AC excitation field with a DC bias field. Our findings demonstrate that under conditions of reduced coil turns and weak excitation fields, the DC bias field exhibits exclusive dependence on the axial distance of the detection point, independent of particle concentration. This implies that the saturated DC bias field corresponding to the second harmonic signal can be used to determine the magnetic nanoparticle sample detection depth. The experimental results validated the method’s high accuracy, with axial detection distance and concentration reduction errors of only 4.8% and 4.1%, respectively. This research paves the way for handheld probes capable of tomographic tracer detection, offering a novel approach for advancing magnetically sensitive biomedical detection technologies.

## 1. Introduction

Magnetic markers consist of a magnetic core encapsulated within a specific biocompatible polymer coating. The magnetic core comprises one or more magnetic nanoparticles (MNPs) [1]. Superparamagnetic iron oxide nanoparticles (SPIONs) are nanoscale ferrimagnetic materials exhibiting superparamagnetic and surface-modifiable properties. These particles generate a rapid, hysteresis-free response to applied magnetic fields, enabling instantaneous detection of the magnetic signal by coils [2,3,4]. Consequently, SPIONs hold promise for developing novel techniques, particularly in magnetic particle imaging (MPI). MPI is a tomographic imaging modality that utilizes SPIONs as contrast agents. Tracer localization is achieved by constructing a zero field point or line spatial scan within a gradient field, requiring strong gradient magnetic fields to enhance spatial resolution [5,6,7]. However, the generation of high-intensity gradient fields necessitates large electromagnetic coils, limiting the applicability of MPI in certain clinical scenarios demanding compact devices, such as lymph node localization and minimally invasive surgery.

In 2013, Shiozawa et al. [8] introduced a novel technique for sentinel lymph node biopsy (SLNB) using superparamagnetic iron oxide nanoparticles (SPIONs) as tracers. This method enabled the detection of 0.03 mL of SPIONs within capillaries up to 20 mm away using a permanent magnet magnetometer. Since then, magnetic detection has become widely adopted for SLN localization. SLNB is used to assess cancer metastasis in lymph nodes and avoid unnecessary lymph node dissection, a procedure that can lead to side effects due to lymphatic drainage obstruction [9,10,11,12,13,14]. Sentinel lymph nodes, the first recipients of lymphatic fluid draining from the primary tumor site (potential location of initial metastasis), are identified using specialized detectors and tracers. Building on this work, the SentiMAG Experimental Centre developed the Sentimag detection device and the Sienna+ magnetic tracer in 2014 [15,16]. Douek et al. [15,16] compared magnetic and standard tracers (radioisotopes or blue dyes) in 160 breast cancer patients undergoing SLNB. They found that the magnetic technique offered a higher detection rate but with a limited maximum depth of approximately 10 mm. However, current magnetic detection methods using fundamental signal acquisition and non-differential sensors are highly susceptible to external interference (e.g., surgical instruments and power supply noise) and lack the ability to support tomographic localization, a crucial feature for intraoperative settings with stringent environmental demands [17,18,19]. Othman et al. [20] proposed using second harmonic detection in MPI for nanoparticle localization in 2015. They employed a singular value decomposition (SVD) analysis of the magnetic field signal to determine the MNP density distribution. This method could detect a 100 μg solid MNP sample 30 mm below the coil and differentiate between two samples separated by 20 mm at that depth. However, it requires rapid scanning of the magnetic field over the imaging area, placing high demands on the excitation field circuitry for achieving high temporal resolution for image reconstruction. Kuwahata et al. [21] (2019) used a permanent magnet excitation probe with Hall element detection to achieve a longitudinal detection distance of 10 mm for 140 μg SPIONs. However, this technique suffers from limitations in depth and sensitivity due to the use of permanent magnets and Hall/magnetoresistive sensors. In 2020, Tanaka et al. [22] developed a laparoscopic AC/DC magnetic probe with a static-to-alternating magnetic field ratio of 5. This design, combining a coil and permanent magnet, could identify 140 μg samples at 10 mm and detect 280 ng samples at 1 mm. Differential coils are commonly used in MPI to mitigate the effects of excitation fields and improve the signal-to-noise ratio. Morishige et al. [23] (2014) employed a differential detection coil to detect a 9 kHz third harmonic signal under a 3 kHz, 1.6 mT excitation field, successfully obtaining a clear particle contour map. Similarly, Bai et al. [24] (2015) designed a narrowband MPI study coil with a gradiometer structure by connecting offset coils in series with a pickup coil. This configuration achieved a baseline of 30 mm and detected two MNP samples with 10 mm spacing and 5 mm spatial resolution at a depth of 35 mm.

In this study, by establishing a gradient-free field, based on the magnetic nanoparticle second harmonic [25,26,27,28,29,30] magnetization signal detection system using a differential structure detection coil, which has the advantage of counteracting the interference of the excitation signal with the detection signal, the interference effect of the excitation field can be suppressed to the maximum extent so as to make the target response signal more prominent. We linearly superimpose the DC bias field under the basis of a constant AC field, determine the AC/DC specific proportionality relationship of the second harmonic maximization when the DC bias field is coupled with the AC magnetic field, analyze the nonlinear magnetization response characteristics of superparamagnetic nanoparticles and the dependence on the DC bias field under different orientations of the distance sensors, and establish an analytical model of the bias field with the distance detection and the real concentration. This model enables the one-dimensional laminar detection of lymph node direction, synchronously determining tracer depth and concentration and significantly enhancing the accuracy of anterior lymph node magnetic detection.

## 2. Theoretical Analysis

### 2.1. Second Harmonic Response Analysis of Magnetic Nanoparticles

Neglecting magnetic particle anisotropy, particle–particle interactions, and hysteresis, the nonlinear magnetization behavior of superparamagnetic iron oxide nanoparticles can be approximated by the Langevin paramagnetic theory. When exposed to an external magnetic field, the magnetic moments of these particles align with the field direction, resulting in a change in magnetization. For ideal particles, magnetization increases gradually with increasing field strength, reaching saturation, and subsequently decreases to zero as the field is removed. This behavior can be mathematically modeled using the Langevin function [28].

(1) $$\frac{\langle m\rangle }{m}=L\left(\frac{mH}{k_{B}T}\right)$$

The average magnetic moment in the direction of the applied magnetic field, denoted by ⟨*m*⟩, is determined by the magnetic moment of the nanoparticles (*m*), the applied magnetic field strength (*H*), the absolute temperature (*T*), and the Boltzmann constant (*k_B_*).

**Figure 1:**
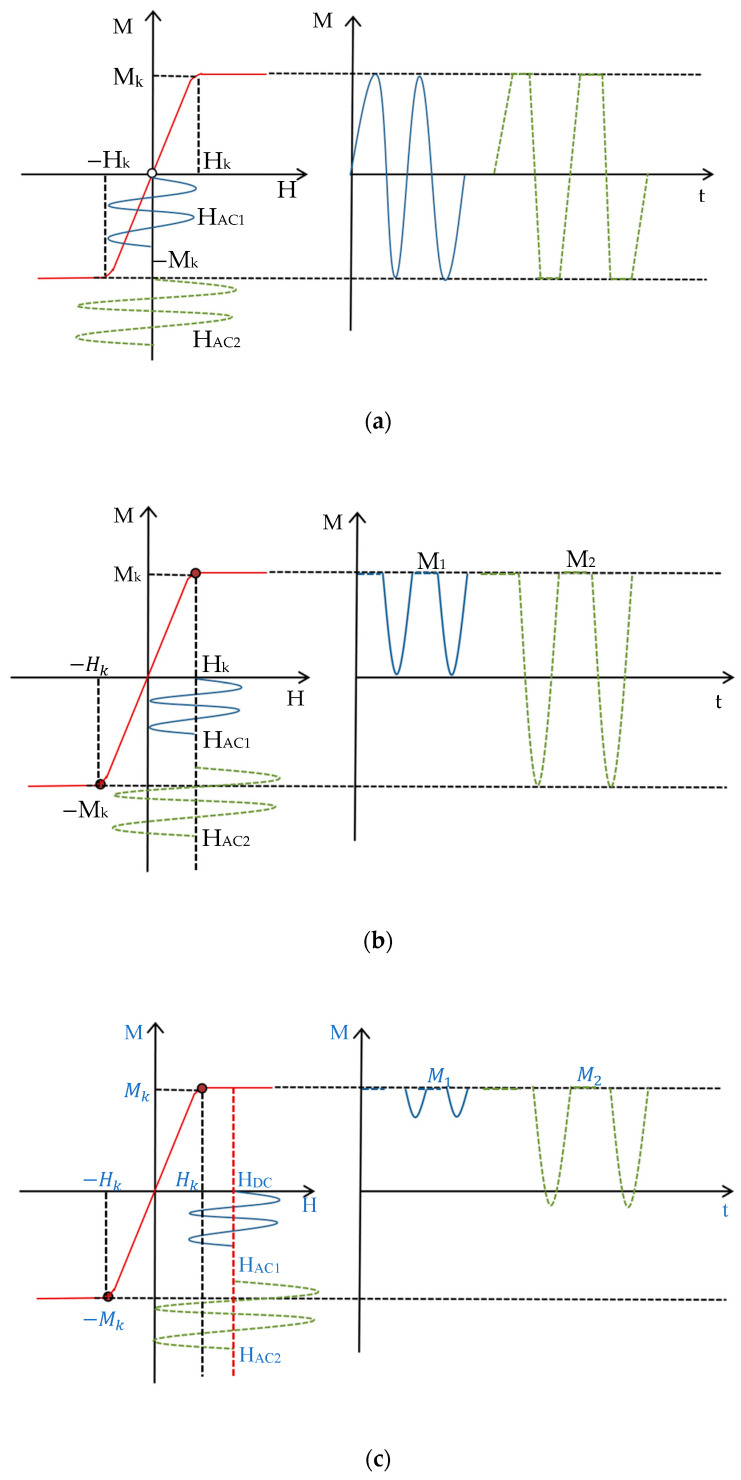
Magnetic nanoparticle harmonic response detection principle. (**a**) Third harmonic response curve under H_AC_ and H_DC_ = 0; (**b**) Third harmonic response curve under H_AC_ and H_DC_ = H_k_; (**c**) Third harmonic response curve under H_AC_ and H_DC_ > H_k_.

Figure 1 shows the schematic diagram of magnetic nanoparticles based on different harmonics detection: *M*-*H* is the nonlinear magnetization response curve of the magnetic nanoparticles, *M* is the magnetization signal, *M_k_* is the saturation magnetization signal, *H* is the applied magnetic field strength, *H_k_* is the applied magnetic field strength required for the magnetic nanoparticles to reach the saturation magnetization signal, *M*-*t* is the time-domain graph of the magnetic nanoparticles’ magnetization signals, and M1 and M2 are the magnetization response of the magnetic nanoparticles at the same frequency with different amplitudes. In particular, Figure 1a represents the principle of MNP detection using the third harmonic response. If a small amplitude AC magnetic field ($H_{AC1}$) is applied, the magnetization response is sinusoidal and contains the fundamental frequency; the magnetization response obtained by applying a large-amplitude AC magnetic field ($H_{AC2}$) is distorted and contains the third and odd harmonics. By attaching a DC bias field $H_{DC}$ to the AC magnetic field, as shown in Figure 1b, the AC field is shifted, and the magnetization response of the magnetic nanoparticles is a half-rectified waveform, which generates even harmonic signals, mainly the second harmonic. When $H_{DC}=H_{k}$, the second harmonic signal of the magnetic nanoparticles reaches the maximum value due to the nonlinear characteristics of its magnetization signal; when $H_{DC}>H_{k}$, the rectified waveform of the magnetic particles’ magnetization response time-domain graph becomes smaller, as shown in Figure 1c, and the time-domain curve tends to be saturated gradually with the second harmonic acceptance signal being zero as $H_{DC}$ increases. As can be seen from Figure 1, based on the second harmonic, the magnetic particle magnetization signal is not affected by the AC field amplitude. Even if the AC field amplitude is small, the particle magnetization response can be observed.

The red curve on the left side of the figure shows the nonlinear magnetization response curve of the magnetic nanoparticles; the small amplitude (H_AC1_) sinusoidal AC voltage waveform is shown in blue, and the green dotted line is the large amplitude (H_AC2_) sinusoidal AC voltage waveform. The curves on the right side of the figure are the time-domain graphs of the magnetic nanoparticle magnetization signals at the same frequency with different amplitudes.

The application of a DC bias to the alternating magnetic field results in a total applied magnetic field *H* that is the sum of the excitation field and the DC bias field.

(2) $$H=\sqrt{2}H_{AC}\cos\left(\omega t\right)+H_{DC}$$

Substitute into Equations (1) and (2):

(3) $$\frac{\langle m\rangle }{m}=coth\left[\frac{\sqrt{2}H_{AC}\cos\left(\omega t\right)+H_{AC}}{k_{B}T/m}\right]-{\left[\frac{\sqrt{2}H_{AC}\cos\left(\omega t\right)+H_{DC}}{k_{B}T/m}\right]}^{-1}$$

The magnetization of the *k*-th harmonic signal is as follows [30] (where the exponent *i* is the first *i* particle signals summed over the magnetized intensity of the kth harmonic signal):

(4) $$M_{k}=\frac{1}{\mu_{0}V_{T}}\sum_{i}n_{i}m_{i}^{2}\frac{{\langle m\rangle }_{ki}}{m_{i}}∆m_{i}$$

**Figure 2:**
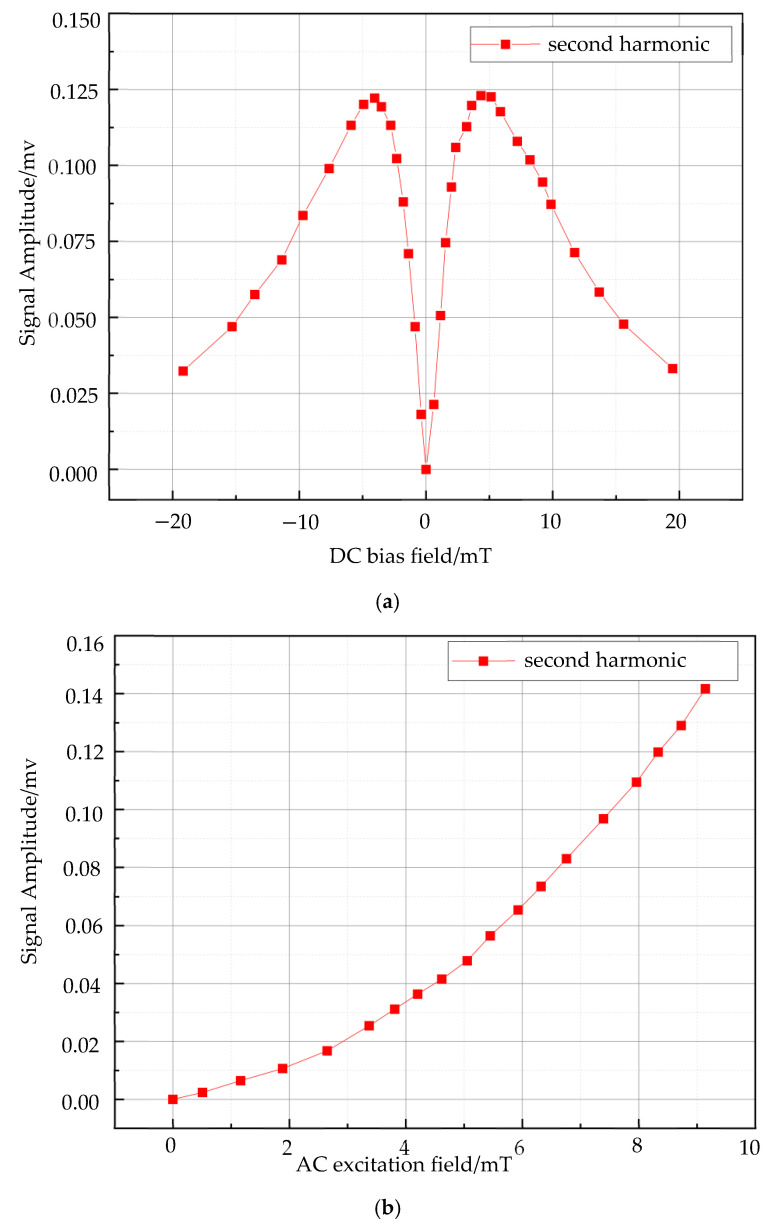
Dependence of the second harmonic signal of MNPs on the AC and DC bias field. (**a**) Effect of DC field on second harmonic signals; (**b**) Effect of AC field on second harmonic signals.

The magnetized signal *M* is expanded by the Taylor series and decomposed into the harmonic signal amplitude intensity *A* (signal peak) of each order, where the second harmonic amplitude intensity $A_{2}$ (signal peak) can be expressed as follows:

(5) $$A_{2}=NM_{s}\left[\frac{H_{AC}^{2}H_{DC}}{30}{\left(\frac{M_{s}}{K_{B}T}\right)}^{3}-\left(\frac{{H_{AC}^{4}H}_{DC}}{189}+\frac{2H_{AC}^{2}H_{DC}^{3}}{189}\right){\left(\frac{M_{s}}{K_{B}T}\right)}^{5}+\cdots \right]$$

where *N* represents the concentration, *M_s_* is the saturation magnetization of the magnetic nanoparticles (MNPs), *H_AC_* is the AC field strength, *H_DC_* is the DC field strength, *k_B_* is the Boltzmann constant, and *T* is the absolute temperature. Given the known values for the MNP concentration, Boltzmann constant, temperature, and saturation magnetization of the sample material, the dependence of the second harmonic signal on the DC and AC excitation fields is illustrated in Figure 2. As shown, the second harmonic signal of the MNPs increases rapidly with the DC bias field until it reaches a peak and subsequently declines. This behavior stems from the nonlinear magnetization characteristics of MNPs. Furthermore, increasing the AC excitation field drives the magnetization signal from the linear regime to the saturation regime.

As depicted in Figure 1, the introduction of a DC bias field shifts the AC excitation field. When 0 < *H_DC_* < *H_k_*, the magnetization curve exhibits asymmetric distortion in the time domain, resulting in an increased induced electromotive force (EMF). At *H_DC_* = *H_k_*, the magnetization response becomes a half-rectified waveform, yielding a maximum second harmonic signal. When *H_DC_* > *H_k_*, the time-domain magnetization signal exhibits small-amplitude fluctuations, ultimately approaching saturation (*M* = *M_s_*) and resulting in a null induced EMF.

### 2.2. Establishing the Relationship between Bias Field and Sample Spatial Distance Based on Langevin Equation

**Figure 3:**
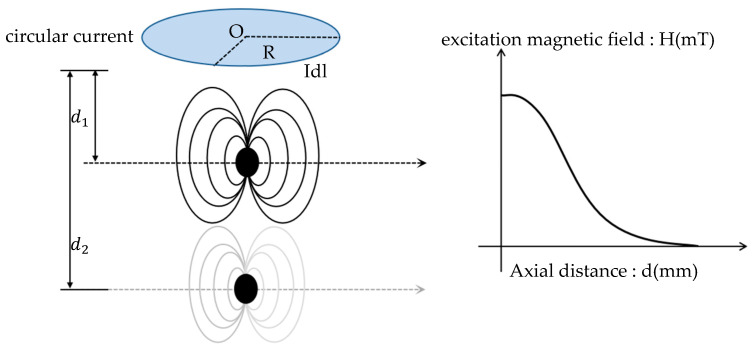
Axial excitation magnetic field distribution.

In practical applications, the excitation magnetic field is typically generated by a solenoid, which can be approximated as a superposition of the magnetic fields produced by a finite number of toroidal coils. While a toroidal coil generates its maximum magnetic field at its center, the sample is often positioned at a distance from the excitation coil, resulting in a weaker magnetization. The magnetic field distribution is illustrated in Figure 3.

**Figure 4:**
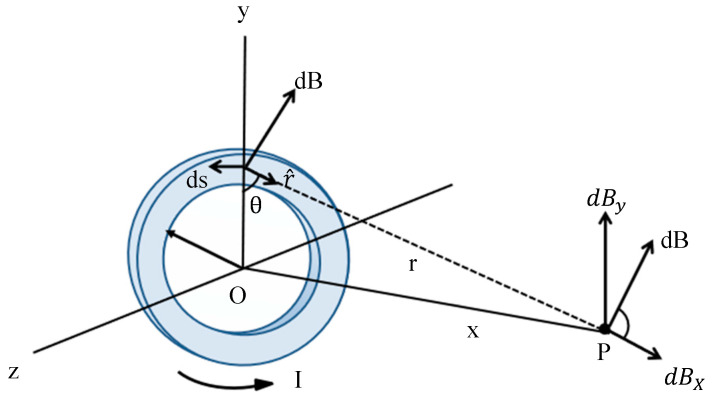
Spatial distribution of magnetic field at point P.

It is assumed that the toroidal coil is on the *xyz*-axis coordinate system, the center of the coil coincides with the coordinate origin O, and the radius of the coil is R; ds is the element per unit of length, r is the distance from the length element to the detection point P, and $\hat{r}$ is the vector of r. The magnetic field at the detection point P must be along the *x*-axis, i.e., (x, 0, 0). Due to the axial symmetry, the dB_y components of the magnetic field cancel each other, and the synthetic field at point P must be along the *x*-axis, i.e., (x, 0, 0). Figure 4 shows the analysis of the magnetic field and the coordinate system of the detection point P on the axis.

According to the Biot–Savart Law:

(6) $$dB=\frac{\mu_{0}I}{4\pi }\frac{\vec{ds}\times \hat{r}}{r^{2}}=\frac{\mu_{0}I}{4\pi }\frac{ds}{\left(x^{2}+R^{2}\right)}$$

(7) $${\;B}_{x}=\oint dB\cos\theta =\frac{\mu_{0}I}{4\pi }\oint \frac{ds\cos\theta }{\left(x^{2}+R^{2}\right)}=\frac{\mu_{0}IR}{4\pi {\left(x^{2}+R^{2}\right)}^{\frac{3}{2}}}\oint ds$$

Substituting ∮ds = 2πR into the preceding equation yields the following expression for B_x_:

(8) $$B_{x}=\frac{\mu_{0}IR^{2}}{2{\left(x^{2}+R^{2}\right)}^{\frac{3}{2}}}$$

Equations (1)–(8) demonstrate that as point P moves along the axial direction (increasing x), the induced magnetic field decreases. The total magnetization of the magnetic nanoparticles is given by

(9) $$\langle M\rangle =NmL\left(\xi \right)$$

where ξ = βmB, *N* refers to the total number of magnetic particles, β = 1/*k_B_T*.

(10) $$L\left(\xi \right)\approx \frac{\xi }{3}-\frac{\xi^{3}}{45}+\frac{2\xi^{5}}{945}+O\left(\xi^{9}\right)$$

**Figure 5:**
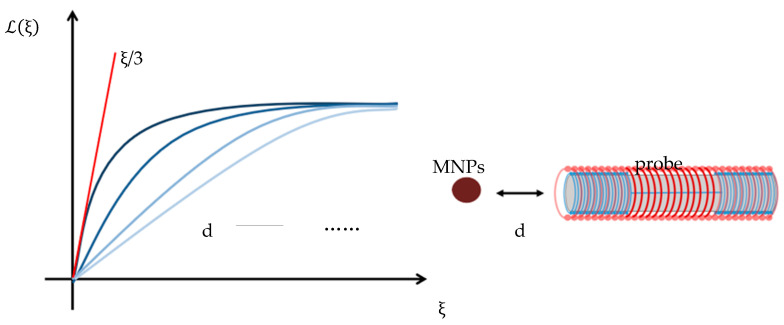
Modeling of Langevin’s function at different detection distances.

Figure 5 depicts the Langevin function as a function of ξ = βmB. As the detection distance d increases, the induced magnetic field B decreases, resulting in a decrease in the slope of the function ξ/3. Consequently, a larger DC bias field is required to saturate the second harmonic magnetization signal of the magnetic nanoparticles.

### 2.3. Probe Structure Design

**Figure 6:**
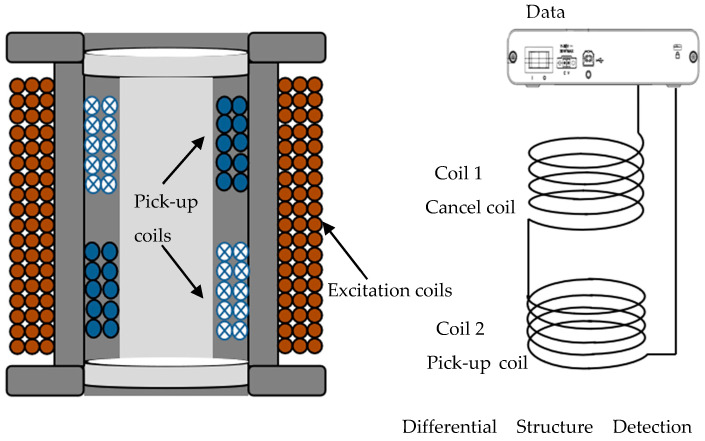
Schematic diagram of probe structure (the left side is a cross-section of the probe structure; the right side is a schematic diagram of the differential structure of the detection coil).

To minimize sensor volume and shield the detection signal from interference generated by the excitation coil, surrounding circuits, and external DC magnetic fields, an anti-interference differential coil structure is employed, as shown in Figure 6. The outermost layer contains the excitation coil, responsible for generating the magnetic field used to stimulate the nanoparticles. The inner layer consists of two detection coils, wound with an equal number of turns but in opposite directions, connected in series for differential mode operation. This configuration maximizes the extraction of the signal from the sample. The nested coil structure effectively cancels the in-phase induced potential from the excitation field within the detection coils, significantly reducing noise and enhancing the signal-to-noise ratio (SNR).

The particle generates an induced magnetization signal under the action of an applied excitation magnetic field, and the receiving coil receives the particle magnetization signal. Wherein coil 1 senses the excitation magnetic field signal *u*_1_, coil 2 senses the particle signal uc superimposed with the excitation magnetic field signal *u*_2_, and the coil 2 and coil 1 differential signal is

(11) $$u=u_{2}-u_{1}+u_{c}$$

Since the coil 1 and coil 2 currents are in opposite directions and in series with each other, then

(12) $$u_{2}-u_{1}=0$$

The detection signal is obtained as

(13) $$u=u_{c}$$

The detection coil is constructed using 790 turns of 1 mm diameter Litz wire, with a 30 mm separation between the two coil sections. Operating at a frequency of 8642 Hz, the coil exhibits an inductance of 3.74 mH and a resistance of 31.57 ohms. To amplify the second harmonic signal at 17,284 Hz, a resonant capacitor is connected in parallel with the detection coil, forming an LC parallel resonant circuit. This circuit enhances the voltage of the signal at the resonant frequency. The value of the resonant capacitance is calculated using the following formula:

(14) $$C=\frac{1}{\omega^{2}L}\approx 90.1nF$$

where *ω* represents the angular frequency of resonance and *L* represents the inductance of the detection coil. The quality factor (*Q*) of the resonant circuit is given by

(15) $$Q=\frac{\omega L}{R}=6.433$$

and detection sensitivity is given by

(16) VSBS=QN2S42πfBsBS=14πNSfQ=2388VT

where *V_s_* represents the voltage signal generated across one side of the coil, *S* denotes the cross-sectional area of the coil, and *N* corresponds to the number of turns in the coil.

The probe utilizes a differential detection principle based on a nested coil structure. An alternating magnetic field generated by the excitation coil induces a nonlinear response in the magnetic nanoparticles, which is subsequently detected by the detection coil. The detection coils, positioned symmetrically above and below the sample, are connected in series with reversed polarity, effectively canceling out induced potentials from the excitation field. Conversely, the nonlinear echo signals emanating from the target nanoparticles are detected primarily by a single coil. This spatial arrangement minimizes interference from the excitation field, thereby amplifying the signal from the target nanoparticles.

## 3. Measurement Experiment

The experiments in this paper utilized Resovist (Fujifilm RI Pharma) superparamagnetic iron oxide nanoparticles. These multinucleated nanoparticles have an average particle size of approximately 21 nm, with individual nuclei ranging from 2 to 7 nm in size. Resovist is currently approved for clinical use by the European Medicines Agency and the Pharmaceuticals and Medical Devices Agency of Japan.

**Figure 7:**
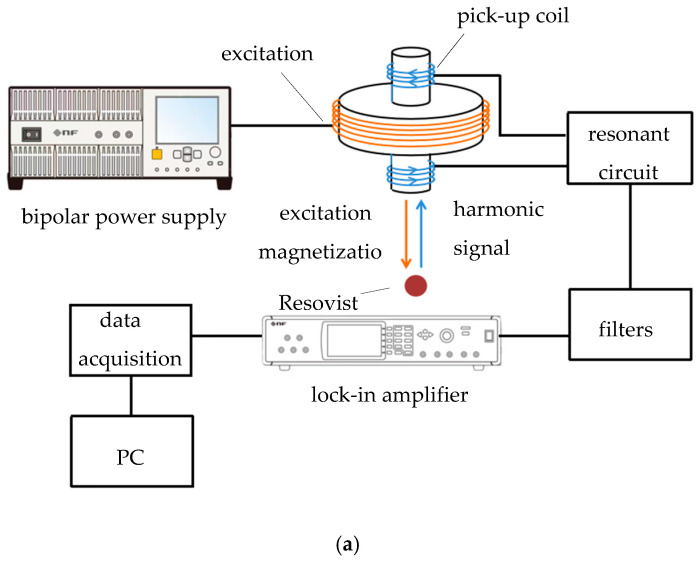

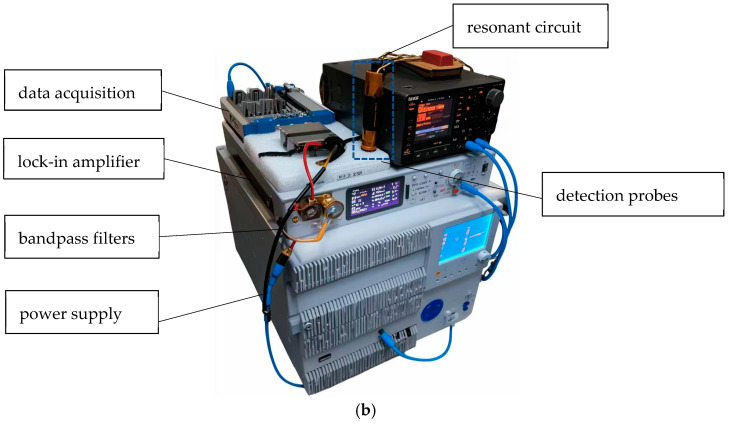
System schematic. (**a**) System block diagram; (**b**) actual picture.

Magnetic nanoparticles exposed to an applied excitation magnetic field generate a magnetization signal that perturbs the surrounding magnetic field. The resulting particle signals are detected by a receiving coil, filtered, and subjected to lock-in amplification to extract the second harmonic signal. Figure 7 illustrates the system’s schematic and physical configuration. An excitation field comprising an AC magnetic field coupled with a DC bias field is applied in the x-direction. Experimentally, a 25 mm diameter excitation coil with 440 turns generated the AC field using a bipolar power supply outputting a constant 21.1 V_P-P_ voltage at 4231 Hz. A DC voltage was linearly superimposed to ensure a magnetic field exceeding 2 mT within a 12 mm radius. The detection coil, a differential structure with a 10 mm diameter, consisted of 790 turns in both the upper and lower sections.

### 3.1. Effect of Sample Concentration on the Second Harmonic Signal of Magnetic Particles

**Figure 8:**
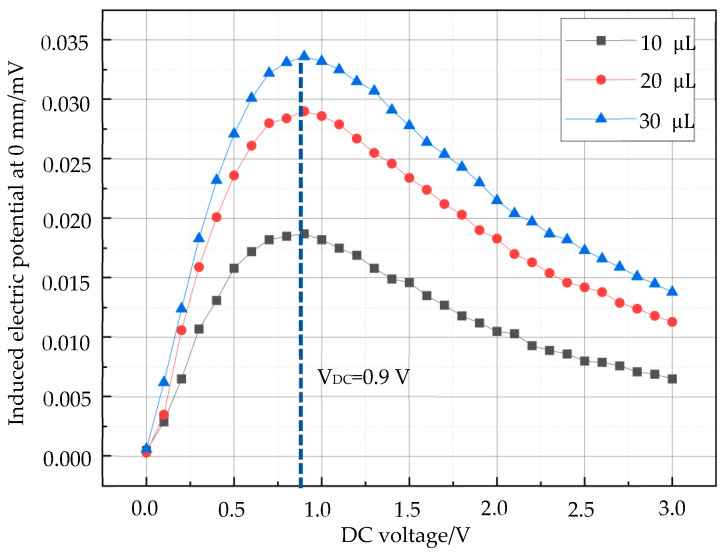
Detection signals of different concentrations of magnetic particles at the same distance.

Keeping the AC voltage constant, the DC voltage was superimposed from 0 V to 3 V at intervals of 0.1 V. The magnetic nanoparticle samples containing 10 μL, 20 μL, and 30 μL of magnetic nanoparticles with a concentration of 44.6 mg/mL (Fe) were detected by the magnetization signal at a distance of 0 mm (from the axial bottom position of the sensor), the second harmonic signals obtained from the detection were recorded, and the experimental results were obtained as shown in Figure 8.

As can be seen from Figure 8, when the axial distance between the magnetic nanoparticle sample and the probe is kept consistent, the peak value of the detection signal is linearly and positively correlated with the particle content, but the DC bias voltage corresponding to the peak value is not related to the particle content. Therefore, according to Equations (1)–(5), when both the AC excitation field and DC bias voltage are constant, the MNP content can be obtained from the peak of the detection signal.

### 3.2. Effect of Sample Distance on the Second Harmonic Signal of Magnetic Particles

A constant AC voltage was maintained, and a DC bias voltage was superimposed in 0.1 V increments from 0 V to 3 V. Magnetization induction signals were recorded at axial distances of 0 mm, 2 mm, 4 mm, 7 mm, and 12 mm from the sensor for SPION samples with concentrations of 30 μL and 20 μL.

**Figure 9:**
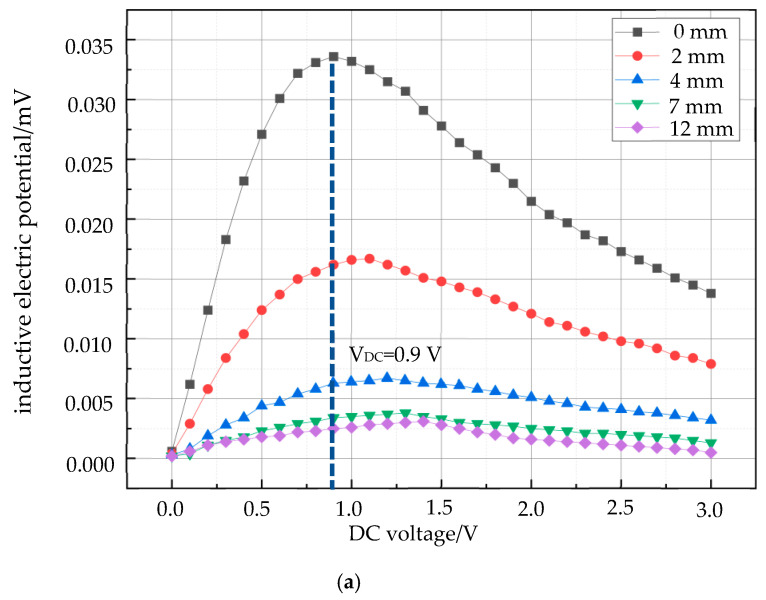

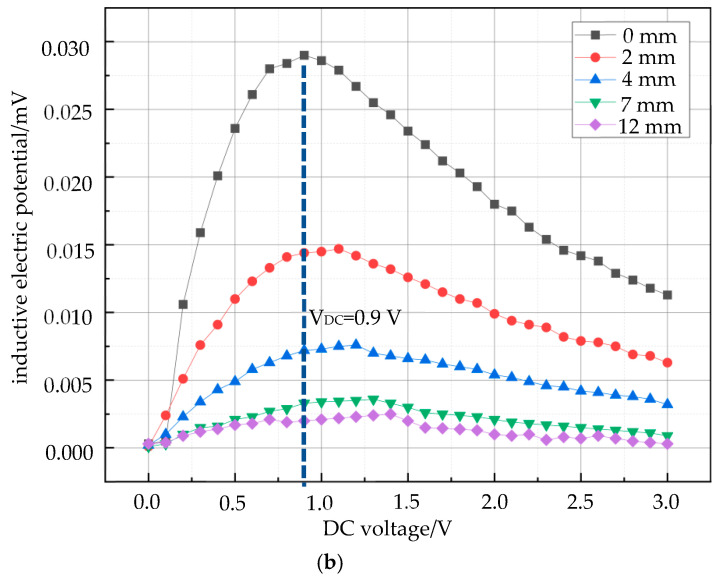
Second harmonic detection signals of magnetic nanoparticles at different distances for 20 μL and 30 μL. (**a**) 30 μL magnetic particle detection signal; (**b**) 20 μL magnetic particle detection signal.

**Figure 10:**
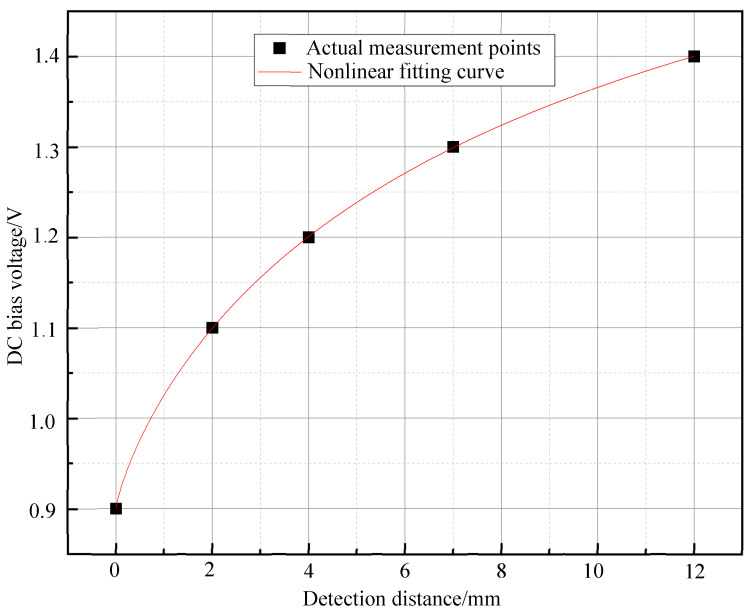
Fitting curve between the DC bias voltage (corresponding to the peak value of the detection voltage) and the detection distance.

Figure 9 illustrates the correlation between increasing the probe–sample axial distance and the required DC bias field for achieving the peak detection signal. As predicted by the Langevin function (Figure 5), the magnetic field at the detection point decreases with distance, while the sample’s magnetization saturation remains constant. Consequently, a larger DC bias field is necessary to induce saturation in the magnetic nanoparticles. Notably, the DC bias field required for the peak signal is independent of nanoparticle concentration. To analyze the relationship between the DC bias field and detection depth, the detection distance was treated as the dependent variable. The data were fitted to a logistic function, yielding a nonlinear correlation coefficient (*R*^2^) of 0.99999, and the functional expression is *V_DC_* = $1.23546-0.93546/\left[1+{\left(\frac{d}{10.1}\right)}^{0.81}\right]$. Figure 10 provides a schematic diagram of this relationship.

### 3.3. Experimental Verification

In order to verify the accuracy of the backpropagation distance and concentration, 30 μL concentrations of 37.125 mg/mL magnetic nanoparticles were selected for the validation experiments. Keeping the AC voltage constant and linearly superimposing the DC source with a step value of 0.05, the detection signal was collected at a distance of 5 mm from the probe in the sample, and the magnetization signal of magnetic nanoparticles peaked at *V_DC_* = 1.25 v. This was substituted into the above distance formula [*d*(*V_DC_*) = 10.1 ${\left(\frac{1.23546-V_{DC}}{V_{DC-0.3}}\right)}^{0.81}$] and concentration formula [$C(V_{DC})\approx A_{2}/\{M_{s}\nu \left[\frac{H_{AC}^{2}\frac{NV_{DC}}{Zl}}{30}{\left(\frac{M_{s}}{K_{B}T}\right)}^{3}-\left(\frac{H_{AC}^{4}\frac{NV_{DC}}{Zl}}{189}+\frac{2H_{AC}^{2}{\left(\frac{NV_{DC}}{Zl}\right)}^{3}}{189}\right){\left(\frac{M_{s}}{K_{B}T}\right)}^{5}\right]\}$], where ν is the sample volume, *N* refers to the number of coil turns, l represents the coil length, and *z* denotes the impedance] to obtain the axial distance d ≈ 5.24 mm and the concentration *C* ≈ 39.7 mg/mL.

The actual detection distance was 5 mm, with an error of 4.8%, and the error of concentration reduction was 6.48%. A number of experiments were conducted to obtain the reduction results and error values for the distance and concentration in Table 1. To validate the accuracy of the backpropagated distance and concentration, experiments were conducted using 30 μL concentrations of 37.125 mg/mL magnetic nanoparticles. The AC voltage was maintained constant, while the DC voltage was linearly incremented in 0.05 V steps. The detection signal was acquired at a 5 mm distance from the probe within the sample. Upon determining the DC bias voltage *V_DC_* = 1.25 V corresponding to the peak magnetization signal, the aforementioned distance and concentration formulas were applied to calculate the axial distance d ≈ 5.24 mm and the concentration of 39.7 mg/mL. The calculated values were compared to the actual values, yielding a 4.8% distance error and a 6.48% concentration reduction error. Table 1 and Table 2 summarizes the results of multiple experiments, including calculated distance and concentration values, as well as their respective errors.

## 4. Discussion

This paper takes magnetic particle imaging as the theoretical basis, aiming to solve the problem of the magnetic nanoparticle tracer based on the difficulty of accurate positioning at biological foci, and investigates the nonlinear distribution of the axial induced magnetic field and the change rule of the second harmonic response signal of magnetic nanoparticles with the bias field. Firstly, the second harmonic signals of magnetic nanoparticles were detected at a distance of 0 mm from the probe using samples with concentrations of 44.6 mg/mL at 10 μL, 20 μL, and 30 μL, respectively, as can be seen in Figure 8. The second harmonic signals of the magnetic nanoparticles with different contents all reached the peak value under the same DC voltage, and the higher the content of the samples, the greater the peak value of the signals, which is in accordance with the theoretical Equation (5), i.e., the peak value of the AC voltage and the detected signal is the same as that of the bias field, which is in accordance with theoretical Equation (5). That is, the detection signal is positively correlated with the number of magnetic nanoparticles when the AC voltage and the corresponding DC voltage at the peak of the detection signal are constant.

Next, we used 20 μL and 30 μL samples to carry out the distance detection experiments under the conditions of 0 mm, 2 mm, 4 mm, 7 mm, and 12 mm from the probe, and the results are shown in Figure 9 and Figure 10, respectively. From the figures, it can be seen that the experimental results are consistent even when using samples with different contents of magnetic nanoparticles, and it can be seen that the DC bias field corresponding to the peak of the detected signal is independent of the number of magnetic nanoparticles. When the distance between the sample and the detection coil increases, the DC voltage corresponding to the peak of the second harmonic detection signal increases, and then the DC bias field at the peak of the signal is nonlinear with the axial distance. Further, when the detection distance is used as the dependent variable in the experiment, the variation in the DC bias field with respect to the detection depth can be obtained.

Finally, we verified the accuracy of the experiment by experimentally inverting the relationship between concentration and distance. As can be seen from Table 1, in this study, for samples with the same content of different concentrations as well as different distances, the calculated and actual errors are all within 10 per cent (within the allowable range of experimental error).

Regarding the heat generation of magnetic nanoparticles in an alternating magnetic field, according to the relevant literature [31], the heat generation rate of magnetic nanoparticles is mainly related to the frequency and the magnetic field strength, and the magnetic nanoparticles generally need to be heated up under the action of a high-frequency and high-intensity alternating magnetic field (50 KHz < f < 1 MHz, 6 mT < H < 62.5 mT) for a long period of time (10–30 min). The excitation frequency used in this experiment is only 4321 Hz, which corresponds to an alternating magnetic field strength of 2 mT, and is only applied to the magnetic nanoparticle signal transient (<10 s) detection process, so the magnetic nanoparticles do not have a significant temperature change in a short period of time, which will not affect the experimental results obtained.

There is still more room for this study to be developed, and to this end, we propose several constructive ideas as follows:

(1) Improvement of positioning accuracy. Although the gradient-free detection system significantly reduces the volume and energy consumption, there is still a certain decline in spatial resolution compared with traditional MPI. In the next step, we need to continue to optimize the design of the detection coil and the signal measurement processing in order to further improve the positioning accuracy and reach the level required for clinical applications.
(2) Improve the anti-interference ability. Since the gradientless system is more sensitive to environmental magnetic interference, the hardware architecture and signal processing algorithms need to be further optimized to ensure the system’s good working ability in complex environments.
(3) Based on the distance–concentration bivariate analytical model, there is still a certain measurement error, which may originate from the DC source superposition step value being too large, resulting in the saturated DC bias field corresponding to the peak point of the signal being not accurate enough. Therefore, the detection sensitivity can be improved by reducing the DC bias field step size.
(4) Different SPIONs have different saturation magnetization curves due to their material properties, and the correlation model between the DC bias field and the spatial axial distance needs to be further optimized to accurately determine the detection depth and concentration of the magnetic nanoparticle tracer.

## 5. Conclusions

By analyzing the second harmonic response signal within a non-gradient field, a correlation was established between the saturated DC bias field corresponding to the second harmonic peak and axial distance. This analysis leveraged the dual nonlinear characteristics of the weak excitation field’s spatial distribution and the Langevin function. A vertical differential coil-based excitation detection probe was designed to accurately locate magnetic nanoparticle tracers spatially. To validate the proposed theory, experimental superposition and coupling of weak excitation AC and linear DC bias fields were employed to detect harmonic signals from SPIONs with varying concentrations and distances. The results indicated that the DC bias field at the signal peak was independent of particle concentration but directly proportional to axial distance. This finding aligns with the theoretical predictions, where increasing axial distance necessitates a corresponding increase in the bias field. Subsequently, an allometric function was established to correlate axial distance and the DC bias field, enabling non-invasive axial distance measurement for unknown depth samples. This lays a foundation for magnetic nanoparticle tracer detection and spatial localization. Future research will focus on model optimization, enhancing axial distance measurement accuracy, and clinical application of the findings.

## Figures and Tables

**Table 1:** Calculation error for 30 μL of 44.6 mg/mL sample at different distances.

Actual Distance /mm	Extrapolated Distance /mm	Distance Error
10	9.57	4.3%
7.5	7.34	2.13%
5	5.24	4.8%
2.5	2.71	8.4%
1	0.96	4.0%

**Table 2:** Calculation error for samples with different concentrations of the same content at 0 mm from the probe.

Actual Concentration $/mg\cdot {mL}^{-1}$	Extrapolated Concentration $/mg\cdot {mL}^{-1}$	Concentration Error
44.6	42.9	3.8%
35.7	37.6	5.3%
26.8	25.9	3.34%
17.6	18.2	3.41%
13.5	12.9	4.48%

## Data Availability

Detailed data are available from the corresponding author upon request.
